# The Association Between Use of Inhaled Corticosteroids and Long‐Acting Beta2‐Agonists During Pregnancy and Adverse Fetal Outcomes

**DOI:** 10.1111/resp.70124

**Published:** 2025-09-09

**Authors:** Yea‐Chwen Wu, I‐Te Wang, Hsin‐Yi Huang, Chung‐Hsuen Wu

**Affiliations:** ^1^ School of Pharmacy, College of Pharmacy Taipei Medical University Taipei Taiwan; ^2^ Department of Pharmacy National Taiwan University Hospital Taipei Taiwan; ^3^ Department of Obstetrics and Gynecology Taipei Medical University Hospital Taipei Taiwan; ^4^ Department of Population Medicine, Harvard Medical School Harvard Pilgrim Health Care Institute Boston Massachusetts USA; ^5^ Department of Pharmacy, College of Pharmaceutical Sciences, National Yang Ming Chiao Tung University Taipei Taiwan

**Keywords:** adverse fetal outcome, asthma, inhaled corticosteroid, long‐acting beta2‐agonist, pregnancy, Taiwan

## Abstract

**Background and Objective:**

Women with asthma should continue controller therapy during pregnancy, but current evidence on the effects of inhaled corticosteroids (ICS) and long‐acting beta2‐agonists (LABA) on adverse fetal outcomes remains unclear.

**Methods:**

This was a population‐based retrospective cohort study. Data were derived from the Health and Welfare Database, Birth Certificate Application, and Maternal and Child Health Database in Taiwan, from January 1, 2007 to December 31, 2018. Pregnant women with asthma were enrolled. Three independent variables included ICS use, ICS dose–response effects, and LABA use during pregnancy. Adverse fetal outcomes included low birth weight, small for gestational age, preterm birth, and congenital anomalies. Propensity score matching (PSM) and inverse probability of treatment weighting (IPTW) were used to adjust for confounders, including sociodemographics, comorbidities, comedications, and asthma severity. Logistic regression models were used to calculate adjusted odds ratios (aORs).

**Results:**

There were 4538 pregnant women with asthma enrolled in this study. After adjustment, neither ICS nor LABA use was significantly associated with any adverse fetal outcomes. However, among women exposed to ICS, high‐dose ICS use during pregnancy was associated with a significantly higher risk of congenital anomalies (aOR: 3.87; 95% CI: 1.29–11.60) within 1 year of delivery.

**Conclusions:**

ICS or LABA use during pregnancy was not associated with the risk of adverse fetal outcomes. Pregnant women with asthma should be advised to maintain controller therapy and avoid potential allergens to reduce the need for high‐dose ICS.

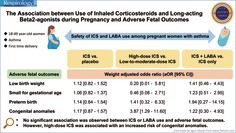

## Graphical abstract

This study updated safety evidence on ICS and LABA use in pregnant women with asthma. After adjusting confounders, there was no association between ICS or LABA use and adverse fetal outcomes. However, high‐dose ICS exposure was associated with a higher risk of congenital anomalies among ICS users.

**See related** editorial

## 1 Introduction

Asthma, the most common respiratory disorder during pregnancy [1], is a risk factor for adverse fetal outcomes. Previous studies showed that pregnant women with asthma were more likely to deliver babies with a low birth weight (LBW), small for gestational age (SGA), and congenital anomalies [2, 3]. Moreover, compared to pregnant women with well‐controlled asthma, those with uncontrolled asthma presented higher risks of a preterm birth (PTB) and intensive care unit admission [4].

Guidelines recommend that women should continue their asthma treatment during pregnancy [5, 6]. Similar to the general population, pregnant women should follow stepwise therapy, using inhaled corticosteroids (ICSs) as first‐line and long‐acting beta2‐agonists (LABAs) as the recommended add‐on. This recommendation was based on evidence that the benefits of using them to reach asthma control may outweigh their threats to fetal health [4, 7, 8, 9, 10, 11, 12, 13, 14, 15].

Medication use during pregnancy requires caution. Although inhaled medications result in relatively low systemic exposure, it remains unavoidable exposure in pregnant women with asthma requiring treatment. In animal models, ICS and LABAs were found to be associated with congenital anomalies through potential interference with fetal organogenesis [16, 17, 18, 19, 20]. Regarding fetal growth, ICS and LABAs may apply opposite effects [21, 22]. ICS use has been linked to potential fetal growth suppression, possibly via endocrine interference [21], whereas LABAs may promote fetal growth by increasing cardiac output and maternal glucose levels [22]. Moreover, the tocolytic use of ritodrine, a beta‐agonist, suggests that LABAs may help prevent preterm birth [23].

Several human studies have evaluated the safety of ICS and LABA use during pregnancy; however, knowledge gaps still remain. First, the existing evidence was reported over a decade ago [8, 9, 10, 11, 12, 13, 14, 15]. Considering changes in treatment recommendations and improvements in prenatal care during these years, updated evidence is needed. Second, some crucial confounding factors–such as asthma severity, comorbidities (e.g., chronic hypertension, and depression), and concomitant medications–were seldom fully adjusted for in previous studies [8, 9, 10, 11, 12, 13, 14, 15]. Third, few studies investigated the safety evidence on the ICS dose–response and add‐on therapy with LABAs [10, 11, 12]. Finally, most previous studies were conducted in Western rather than Asian countries [8, 9, 10, 14]. Whether those findings are applicable to broader populations worldwide remains uncertain.

Therefore, the study aimed to provide updated safety evidence on ICS and LABA use among pregnant women, including ICS use, ICS dose–response effects, and add‐on therapy with LABAs. We used real‐world data from a population‐based administrative database in Taiwan with propensity score to update and enrich the existing evidence. We hypothesised that after addressing confounders, in pregnant women with asthma, ICS use would be associated with higher risks of adverse fetal outcomes; high‐dose ICS use with greater risk than low‐to‐moderate‐dose ICS use; and LABA use with lower risks, except for congenital anomalies.

## 2 Methods

### 2.1 Data Source

The present study used data from the Health and Welfare Database (HWD), the Birth Certificate Application (BCA), and the Maternal and Child Health Database of Taiwan [24]. All information was de‐identified. The HWD contains administrative claims data from the National Health Insurance program, in which over 99% of residents are enrolled [25]. Sociodemographics and patient‐level health service information, including diagnoses, surgeries, and prescriptions, can be obtained [25]. Diagnoses were classified by the International Classification of Diseases, Ninth Revision, Clinical Modification (ICD‐9‐CM) and ICD‐10‐CM. Medication prescriptions were identified by Anatomical Therapeutic Chemical (ATC) codes. Infants born in Taiwan are registered in the BCA, which contains infants' characteristics, such as gender, gestational week, and birth weight [26]. The Maternal and Child Health Database provides linkages between mothers and children [26]. These three databases can be linked by personal identity numbers. The overall data duration covered January 1, 2007 to December 31, 2018.

### 2.2 Study Population

Pregnant women with asthma aged between 18 and 49 years from January 1, 2009 to December 31, 2017 were enrolled. We obtained women's first delivery and excluded women who had any delivery record during 2007–2008 to ensure that they had not given birth for at least 2 years. We used ICD‐CM code to define the diagnosis of asthma. An asthma diagnosis was defined as having at least one inpatient or two outpatient diagnoses (ICD‐9‐CM: 493, excluding 493.2; ICD‐10‐CM: J45) from 90 days before the pregnancy through delivery [27, 28, 29]. Exclusion criteria included multiple births, stillbirths, other chronic respiratory, inflammatory, or immune diseases, adrenal insufficiency, or Cushing's syndrome [30]. Pregnant women who used unrecommended treatment, including chromones, LABAs without ICSs, or long‐acting muscarinic antagonists (LAMAs) without ICSs, were excluded [5]. Women exposed to known teratogenic agents during the first trimester or with chromosome abnormalities were also excluded [9, 31]. Finally, women not continuously enrolled in the insurance programme from 90 days before pregnancy through delivery were excluded to ensure complete information collection.

### 2.3 Exposures

Three separate exposures, ICSs, high‐dose ICSs, and ICSs‐LABAs, were evaluated in order. First, ICS exposure was defined as at least one ICS prescription during pregnancy (ATC codes: R03BA and R03AK); women who were not exposed to ICSs from 90 days before pregnancy through delivery were assigned to the reference group. Second, women with asthma exposed to at least one ICS during pregnancy were enrolled to evaluate the effects of high‐dose ICSs and add‐on LABA therapy. ICS dose classification was defined based on the criteria from the Global Initiative for Asthma (GINA) guidelines [5]. The mean daily dose was calculated in budesonide equivalents and used to divide pregnant women into high‐dose ICS users (> 800 μg) or low‐to‐moderate‐dose users (200–800 μg) [5]. Third, as for LABA exposure, women were assigned to the exposure group if they had at least one prescription of LABAs during pregnancy (ATC codes: R03AC and R03AK); those with no record of LABA use from 90 days before pregnancy through delivery were defined as the reference group.

### 2.4 Outcome Measures

Adverse fetal outcomes included LBW, SGA, PTB, and congenital anomalies. LBW was defined as less than 2500 g; SGA was less than the 10th percentile of the gender‐specific birth weight for gestational age. The 10th percentiles of birth weight were developed using current birth weight data in the BCA with a previously published method [32]. PTB was less than 37 weeks of gestational age. Congenital anomalies were estimated in 1‐year follow‐up periods to avoid delayed diagnoses, defined as at least one inpatient or at least two outpatient diagnoses during this period [33].

### 2.5 Covariates

Covariates were collected within 90 days before pregnancy. Covariates included sociodemographics, maternal comorbidities, comedications, asthma severity, and asthma exacerbations. Sociodemographics were infant gender, maternal age, area of residence at delivery, average insurance premium, and delivery year. Comorbidities involved allergic rhinitis, atopic dermatitis, diabetes mellitus, hypertension, cardiovascular diseases, anxiety, depression, obesity, bipolar disorder, schizophrenia, epilepsy, thyroid disorder, renal disease, antiphospholipid syndromes, anaemia, alcohol abuse, and drug dependence. Comedications were other anti‐asthmatics, systemic corticosteroids, topical corticosteroids, methylxanthine, antihistamines, non‐steroidal anti‐inflammatory drugs, decongestants, antihypertensives, non‐insulin antidiabetics, insulin, antihyperlipidemics, anti‐thrombotics, antidepressants, benzodiazepines, anticonvulsants, antipsychotics, antithyroid or thyroid replacements, opioids, codeine, antibiotics, and folic acid. Asthma severity was defined based on the anti‐asthmatics use and categorised as mild (steps 0, 1, and 2), moderate (step 3), and severe (steps 4 and 5) [5, 27]. The highest severity during the baseline period was used. Asthma exacerbations were defined by asthma‐related emergency department visits, asthma‐related hospitalisations, and a course of oral corticosteroids [27]. Detailed definitions of asthma exacerbations are provided in Table S1.

### 2.6 Statistical Analyses

The propensity score was used to balance covariates between the exposure and reference groups. When evaluating the effects of ICSs and LABAs, one‐to‐one propensity score matching (PSM) was performed by nearest neighbour matching with a calliper width equal to 0.2 of the standard deviation of the logit of the propensity score [34, 35]. Inverse probability of treatment weighting (IPTW) with weight stabilisation was used in the ICS dose–response evaluation due to the small number of high‐dose ICS users [34]. To estimate adjusted odds ratios (ORs; aORs) of adverse fetal outcomes, a conditional logistic regression and weighted logistic regression were conducted after PSM and IPTW, respectively. Standardised mean differences (SMDs) were calculated to assess covariate balance between exposure and reference groups. If covariates were imbalanced (SMD > 0.1) after PSM or IPTW, they were further adjusted for in the logistic regressions [34, 36]. All statistical analyses in this study were generated using SAS software vers. 9.4 (SAS Institute, Cary, NC, USA). aORs were performed with a 95% confidence interval (CI). A two‐tailed *p* value of < 0.05 was considered statistically significant. The study was approved by the Taipei Medical University Institutional Review Board (N202303090).

### 2.7 Sensitivity Analyses

We conducted several sensitivity analyses to test the robustness of the principal findings. First, exposure was redefined as having at least two prescriptions during pregnancy, to preclude occasional exposure. Second, a new‐user design was applied, in which pregnant women who used ICSs or LABAs within 90 days before pregnancy were excluded. Third, pregnancy‐related variables and asthma exacerbations during pregnancy were added to the model as covariates. Pregnancy‐related variables included gestational diabetes, pregnancy‐induced hypertension, eclampsia/preeclampsia, infections during pregnancy, placental abruption, placenta previa, and influenza.

## 3 Results

After selection, 4538 pregnant women with asthma were enrolled (Figure S1). There were 1713 (37.7%) pregnant women with asthma who used ICSs during their pregnancy. Most ICS users used budesonide (1109 [64.7%]), followed by fluticasone (521 [30.4%]). Table S2 shows baseline characteristics before and after PSM. Before PSM, there were differences between ICS users and nonusers, including delivery years, severity of asthma, comorbidity of allergic rhinitis, anti‐asthmatics use (except for omalizumab), topical corticosteroid use, and non‐steroidal anti‐inflammatory drugs use. After PSM, there were 1082 women remaining in each group. All variables were balanced between ICS users and ICS nonusers (absolute SMD < 0.1). Distributions of PS between the two groups also highly overlapped (Figure S2). Baseline characteristics of high‐dose ICS users, low‐to‐moderate‐dose ICS users, LABA users, and LABA nonusers were provided in Tables S3 and S4.

Tables 1, 2, 3 display the results of incidences and adjusted ORs (aORs) of adverse fetal outcomes. Neither ICS nor LABA use during pregnancy was significantly associated with any adverse fetal outcome. However, compared to low‐to‐moderate ICS use, high‐dose ICS use was associated with a significantly increased risk of congenital anomalies (24.3% vs. 7.9%; aOR: 3.87; 95% CI: 1.29–11.60, *p* = 0.02). The risk of LBW, SGA, or PTB did not significantly differ between the high‐dose and low‐to‐moderate‐dose groups.

**TABLE 1:** Incidence and adjusted odd ratios (aORs) of adverse fetal outcomes between inhaled corticosteroid (ICS) users and nonusers.

Outcomes, *N* (%)	Before PSM		After PSM			
	ICS users (*N* = 1713)	ICS nonusers (*N* = 2825)	ICS users (*N* = 1082)	ICS nonusers (*N* = 1082)	aOR [95% CI]	*p*
LBW	160 (9.3)	236 (8.4)	95 (8.8)	86 (7.9)	1.12 [0.82–1.52]	0.48
SGA	234 (13.7)	348 (12.3)	140 (12.9)	133 (12.3)	1.06 [0.82–1.37]	0.65
PTB	160 (9.3)	230 (8.1)	93 (8.6)	82 (7.6)	1.14 [0.84–1.54]	0.40
Congenital anomalies	165 (9.6)	256 (9.1)	106 (9.8)	92 (8.5)	1.17 [0.87–1.57]	0.30

Abbreviations: CI, confidence interval; LBW, low birth weight; PSM, propensity score matching; PTB, preterm birth; SGA, small for gestational age.

**TABLE 2:** Incidence and adjusted odds ratios (aORs) of adverse fetal outcomes between high‐dose and low‐to‐moderate‐dose inhaled corticosteroid (ICS) use.

Outcomes, *N* (%)	Before IPTW		After IPTW			
	High‐dose ICS (*N* = 37)	Low‐to‐moderate‐dose ICS (*N* = 763)	High‐dose ICS (*N* = 22.4)	Low‐to‐moderate‐dose ICS (*N* = 767.3)	aOR [95% CI]	*p*
LBW	5 (13.5)	84 (11.0)	0.4 (1.6)	82.9 (10.8)	0.20 [0.01–5.81]	0.35
SGA	5 (13.5)	120 (15.7)	1.4 (6.2)	118.4 (15.4)	0.46 [0.08–2.71]	0.39
PTB	6 (16.2)	76 (10.0)	2.3 (10.1)	76.6 (10.0)	1.41 [0.32–6.33]	0.65
Congenital anomalies	9 (24.3)	61 (8.0)	5.4 (24.3)	61.0 (7.9)	3.87 [1.29–11.60]	0.02

Abbreviations: CI, confidence interval; IPTW, inverse probability of treatment weighting; LBW, low birth weight; PTB, preterm birth; SGA, small for gestational age.

**TABLE 3:** Incidence and adjusted odds ratios (aORs) of adverse fetal outcomes between long‐acting beta2‐agonist (LABA) users and nonusers.

Outcomes, *N* (%)	Before PSM		After PSM			
	LABA users (*N* = 1207)	LABA nonusers (*N* = 479)	LABA users (*N* = 457)	LABA nonusers (*N* = 457)	aOR [95% CI]	*p*
LBW	104 (8.6)	52 (10.9)	38 (8.3)	48 (10.5)	1.41 [0.46–4.43]	0.55
SGA	160 (13.3)	69 (14.4)	59 (12.9)	66 (14.4)	1.23 [0.51–2.95]	0.65
PTB	118 (9.8)	40 (8.4)	39 (8.5)	35 (7.7)	1.94 [0.27–14.15]	0.51
Congenital anomalies	124 (10.3)	39 (8.1)	47 (10.3)	34 (7.4)	1.22 [0.30–4.93]	0.78

Abbreviations: CI, confidence interval; LBW, low birth weight; PSM, propensity score matching; PTB, preterm birth; SGA, small for gestational age.

**FIGURE 1:**
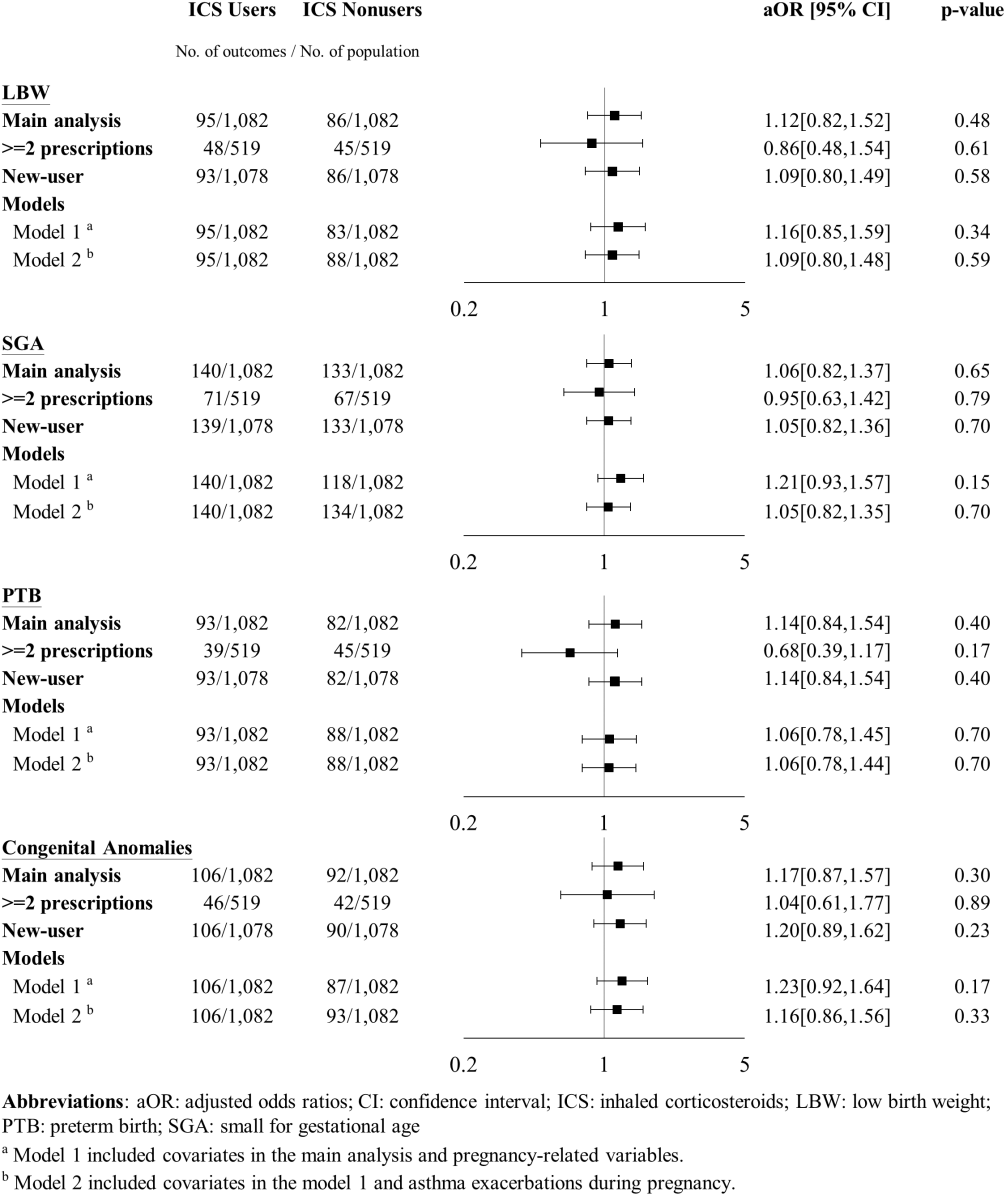
Forest plot of sensitivity analysis of inhaled corticosteroid (ICS) exposure during pregnancy. AOR, adjusted odds ratios; CI, confidence interval; LBW, low birth weight; PTB, preterm birth; SGA, small for gestational age. (a) Model 1 included covariates in the main analysis and pregnancy‐related variables. (b) Model 2 included covariates in model 1 and asthma exacerbations during pregnancy.

**FIGURE 2:**
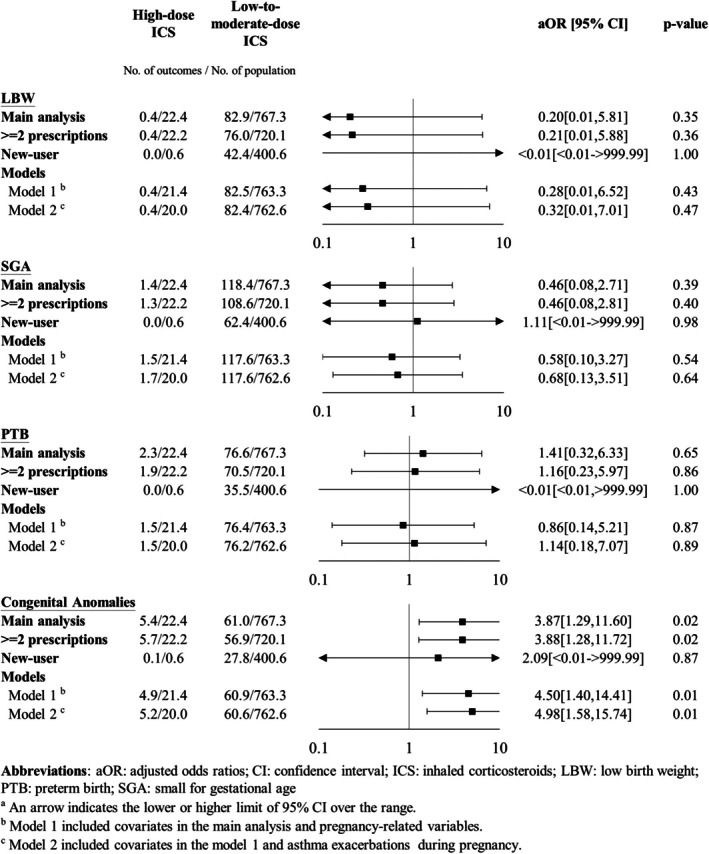
Forest plot of sensitivity analysis of inhaled corticosteroid (ICS) dose–response effects. AOR, adjusted odds ratios; CI, confidence interval; LBW, low birth weight; PTB, preterm birth; SGA, small for gestational age. (a) Arrows indicate the lower or higher limit of the 95% CI over the range. (b) Model 1 included covariates in the main analysis and pregnancy‐related variables. (c) Model 2 included covariates in model 1 and asthma exacerbations during pregnancy.

**FIGURE 3:**
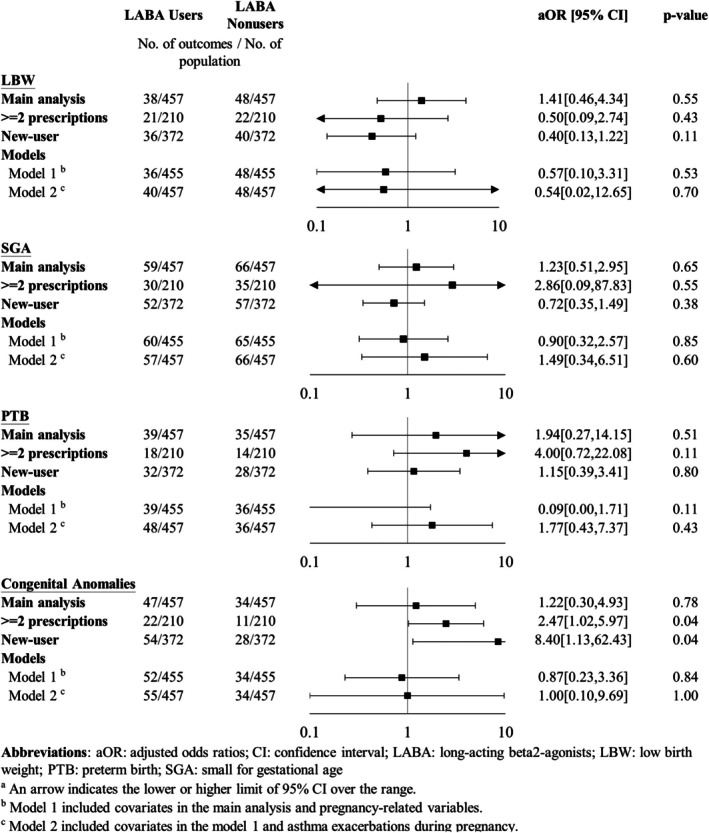
Forest plot of a sensitivity analysis of long‐acting beta2‐agonist (LABA) exposure during pregnancy. AOR, adjusted odds ratios; CI, confidence interval; LBW, low birth weight; PTB, preterm birth; SGA, small for gestational age. (a) Arrows indicate the lower or higher limit of the 95% CI over the range. (b) Model 1 included covariates in the main analysis and pregnancy‐related variables. (c) Model 2 included covariates in model 1 and asthma exacerbations during pregnancy.

Sensitivity analyses showed generally consistent results with the main analyses (Figures 1, 2, 3). The slight difference was that LABAs were associated with a significantly increased risk of congenital anomalies when exposure was defined as at least two prescriptions during pregnancy (10.5% vs. 5.2%; aOR: 2.47; 95% CI: 1.02–5.97, *p* = 0.04) and with the new‐user design (14.5% vs. 7.5%; aOR: 8.40; 95% CI: 1.13–62.43, *p* = 0.04). Although there were statistical differences between LABA users and LABA nonusers in these two sensitivity analyses, *p* values were close to 0.05.

## 4 Discussion

Among pregnant women with asthma, neither ICS use nor add‐on therapy with LABAs during pregnancy was significantly associated with an increased risk of adverse fetal outcomes. However, compared to low‐to‐moderate‐doses ICS use, high‐dose ICS use was significantly associated with an increased risk of congenital anomalies.

Findings in this study that ICS use during pregnancy was not associated with increased risks of adverse fetal outcomes were consistent with previous findings. One meta‐analysis evaluating the association between ICSs and adverse fetal outcomes among pregnant women with asthma observed no increased risk of major congenital anomalies (OR: 0.96; 95% CI: 0.51–1.83), PTB (OR: 0.99; 95% CI: 0.80–1.22), or LBW (OR: 0.89; 95% CI: 0.70–1.14) [37]. In a randomised controlled trial, START, 313 participants with mild‐to‐moderate asthma got pregnant during the follow‐up period. A similar incidence of congenital anomalies between the budesonide group (*n* = 3, incidence: 1.5%) and placebo group (*n* = 4, incidence: 3.4%) was found [38]. Our study population had similar characteristics to participants in the START trial. In this study, after PSM, over 99% of the study population was classified as having mild asthma. Our study findings were similar to those from the START trial but provided updated real‐world evidence with a larger sample size.

Consistent with previous findings, high‐dose ICS use was associated with an increased risk of congenital anomalies. Blais et al. found that, compared with a mean daily ICS dose of <1000 μg beclomethasone equivalents, doses > 1000 μg were associated with a 66% higher risk of any congenital anomalies (aOR: 1.66; 95% CI: 1.02–2.68) [11]. A higher estimated risk was found in our study (aOR: 3.87; 95% CI: 1.29–11.60). This difference might have resulted from the different definitions of exposure period and characteristics of study populations. This study used the entire pregnancy as the exposure period, while Blais et al.'s study only considered the first‐trimester exposure. Women classified as high‐dose users in this study might have had more severe asthma and used higher ICS doses throughout pregnancy. In addition, the study populations in Blais et al.'s and our study differed in characteristics. In Blais et al.'s study, women > 34 years accounted for 6.3%, but in our study, 38.4% were aged ≥ 35 years. Furthermore, the database used by Blais et al. tended to include women of lower socioeconomic status. Mediating effects of these two factors might exist. Lastly, as an observational study, our findings can only demonstrate associations rather than causation. The limited sample size may increase overfitting risk and model instability, reducing generalisability and reliability.

The dose–response effects of ICSs on growth restriction and PTB were not observed in previous studies or our study. Namazy et al. conducted a prospective cohort study of pregnant women with asthma and divided them into four groups by quartiles of daily ICS doses [14]. They observed a trend of increased risk of SGA at higher daily ICS doses, but there was no significant difference among the four groups. Cossette et al. conducted a retrospective cohort study and additionally assessed the ICS dose–response effects on PTB and LBW [10]. Pregnant women with asthma were sub‐grouped by the daily ICS dose, 0, > 0–62.5, > 62.5–125, > 125–250, > 250–500, and > 500 μg in fluticasone equivalents. A trend of increased ORs with an increasing ICS dose was also observed, but there was no significantly increased risk between each dose and the reference group [10]. Although we used different criteria to subgroup ICS users, our study provided similar findings that higher ICS doses were not significantly associated with increased risk of fetal growth restriction or PTB.

The non‐significant association between LABA use and adverse fetal outcomes was similar to previous findings. Vasilakis‐Scaramozza et al. did not observe a significantly lower risk of congenital anomalies (relative risk [RR]: 0.8; 95% CI: 0.4–1.5) among pregnant women with asthma using LABAs compared to healthy pregnant women [9]. Eltonsy et al. found a non‐significantly increased risk of congenital anomalies among pregnant women with asthma who were LABA users (OR: 1.37; 95% CI: 0.92–2.17) [8]. Bracken et al. observed similar risks of PTB (OR: 0.99; 95% CI: 0.97–1.02) and intrauterine growth restriction (IUGR; OR: 1.00; 95% CI: 0.99–1.02) between LABA exposure and the reference group [15]. Cossette et al. found that LABAs were associated with non‐significantly lower ORs of PTB (OR: 0.84; 95% CI: 0.61–1.15), LBW (OR: 0.81; 95% CI: 0.58–1.12), and SGA (OR: 0.92; 95% CI: 0.70–1.20) among pregnant women with asthma [10]. In this study, we additionally restricted our study population to pregnant women with asthma who used ICSs during pregnancy to clarify the effects of LABAs and reduce confounding by indications. Nonetheless, similar to previous studies, our study did not identify a significant association between LABA use and adverse fetal outcomes.

From a biological perspective, two mechanisms may explain our findings. First, inhaled therapies had relatively low systemic exposure, which may be insufficient to induce systemic effects and adverse fetal outcomes. While most human studies have not found an increased risk with ICS or LABA use, high‐dose ICS has been associated with a higher risk of congenital anomalies. In our sensitivity analysis, a borderline increased risk of congenital anomalies was also observed among those with at least two prescriptions of LABA. This may be attributed to elevated serum levels, which could enhance transplacental transfer and potentially affect fetal development [39]. However, current human studies used prescription to define exposure; whether this operational measure correlated with elevated serum levels remains uncertain. Given the borderline *p* value observed in the sensitivity analysis, a type I error cannot be ruled out, and the association warrants further investigation. Second, birth weight could be influenced by multiple factors. Recent studies have not shown a significantly increased risk of LBW or SGA associated with ICS or LABA, even at high‐dose ICS. Corticosteroids impair growth through several mechanisms, such as inhibiting growth hormone secretion and reducing collagen synthesis [40], yet they may also have the opposite effect. For example, hyperglycemia—a known adverse effect of corticosteroids—may lead to increased fetal weight [41]. The confidence intervals were wide in our study, possibly due to the limited sample size. Further investigation is needed to clarify whether ICS has a significant effect on birth weight.

### 4.1 Clinical Implications

Consistent with current guidelines, we recommend that pregnant women with asthma continue ICS or ICS–LABA therapy during pregnancy when clinically indicated, but high‐dose ICS should be used cautiously due to its significant association with congenital anomalies found in our study. Several approaches can assist pregnant women who require high‐dose ICS. First, to reduce high‐dose ICS, pregnant women with asthma should be informed of the importance of preventing acute exacerbations by maintaining controller therapy and avoiding allergens. Second, health care providers can inform women with asthma of the potential risks associated with high‐dose ICS before or early in pregnancy. Finally, monitoring fetal growth closely enables early detection of abnormalities, which can assist in clinical decision‐making and preparation for neonatal care.

### 4.2 Strengths and Limitations

There were several strengths of this study. First, the HWD provided objective information on medication use and disease diagnoses, representative of real‐world experience [42]. Second, the use of PSM efficiently adjusted for several confounders, including disease severity, asthma exacerbations, comorbidities, and comedications. Third, several sensitivity analyses were conducted to consolidate the findings. Last, this study enrolled a large sample size from a national population‐based health insurance database, which represents the population in Taiwan [42].

This study also had several limitations. First, the identification of asthma cases may still be influenced by limitations inherent to the HWD, such as a lack of information for key diagnostic elements for respiratory symptoms and evidence of expiratory airflow limitation. However, we tried to identify asthma based on validated ICD codes and a surrogate algorithm (requiring at least one inpatient or two outpatient diagnoses) supported by prior respiratory disease research [29], which has demonstrated reasonable accuracy for capturing clinically relevant asthma cases in administrative databases. Second, misclassification in defining ICS and LABA exposure is possible. Although exposure was based on prescription records, actual exposure may have varied with adherence and inhaler technique, which were not captured. This potential misclassification may have affected internal validity, particularly in the dose–response analysis with its limited sample size. Third, residual confounding may persist, as key variables such as smoking, BMI, nutritional status, lung function, and oxygen saturation were unavailable in the HWD. Although diagnosis and medication codes were used to serve as proxies (e.g., obesity for overweight and anti‐asthmatics for disease severity), residual bias remains possible because these proxies may not precisely represent patient characteristics. Fourth, the sample size was limited for evaluating the ICS dose–response effect and conducting the new‐user sensitivity analysis. Only 37 women were exposed to high‐dose ICS, which may have led to overfitting and model instability. The findings should be interpreted cautiously due to limited generalisability and reliability. It should also be noted that, as an observational study, this analysis only provided evidence of association rather than causation. Regarding the new‐user design, although the findings were consistent with the main analysis, the small sample size led to a wide confidence interval and reduced precision. Fifth, prevalent user bias might exist. The new‐user design was not used in the main analysis because most pregnant women with asthma may have been diagnosed with asthma and used anti‐asthmatics before their pregnancy. Restricting to new users during pregnancy may have led to a relatively smaller and less representative sample. To reduce the prevalence bias, we adjusted for ICS and LABA use during the baseline period and used a new‐user design as a sensitivity analysis. Results were consistent with the principal findings, meaning that the effect of prevalent‐user bias might have been less significant. Sixth, pregnant women exposed to certain medications in early pregnancy may affect fetal outcomes. However, due to an insufficient sample size, analyses to assess the effect of ICS or LABA use by trimester could not be performed. Finally, selection bias may arise from using a health insurance database. For example, a lack of continuous enrollment could lead to incomplete health information [43]. However, the National Health Insurance program in Taiwan is a government‐run single‐payer system that covers over 99% of the Taiwanese population [25], making data from HWD relatively comprehensive. Consequently, the potential for selection bias was considered low.

In conclusion, in this population‐based retrospective cohort study, ICS or LABA use during pregnancy was not significantly associated with adverse fetal outcomes. However, high‐dose ICS use was associated with an increased risk of congenital anomalies. Pregnant women with asthma should maintain controller therapy and avoid allergens to reduce the need for high‐dose ICS.

## Author Contributions

**Yea‐Chwen Wu:** conceptualization (equal), data curation (equal), formal analysis (equal), investigation (equal), methodology (equal), writing – original draft (equal), writing – review and editing (equal). **I‐Te Wang:** conceptualization (equal), writing – review and editing (equal). **Hsin‐Yi Huang:** supervision (equal), writing – review and editing (equal). **Chung‐Hsuen Wu:** conceptualization (equal), funding acquisition (equal), methodology (equal), project administration (equal), resources (equal), supervision (equal), writing – review and editing (equal).

## Ethics Statement

The study was approved by the Taipei Medical University Institutional Review Board (N202303090). The requirement for informed consent was waived because this was a retrospective observational study using de‐identified data from a healthcare insurance database.

## Conflicts of Interest

The authors declare no conflicts of interest.

## Supporting information


**Data S1:** Supporting Information.

## Data Availability

Data were obtained from the Health and Welfare Database (Taiwan) under license and are not publicly available due to privacy regulations.
