# Supplementary material for: The Association Between Use of Inhaled Corticosteroids and Long‐Acting Beta2‐Agonists During Pregnancy and Adverse Fetal Outcomes
Source: Respirology. 2025 Sep 9;31(1):32–41. doi: 10.1111/resp.70124 (PMC12783959; doi:10.1111/resp.70124)
Supplement: Supplementary file 1 — Data S1: Supporting Information. [file RESP-31-32-s001.docx]

**Supplementary material**

[**Supplementary Table 1.** Definitions of acute asthma exacerbations 2](#_Toc193194921)

[**Supplementary Table 2.** Baseline characteristics of the study population (inhaled corticosteroid (ICS) users vs. nonusers) 3](#_Toc193194922)

[**Supplementary Table 3.** Baseline characteristics of the study population (high-dose vs. low-to-moderate-dose inhaled corticosteroids (ICSs)) 7](#_Toc193194923)

[**Supplementary Table 4.** Baseline characteristics of the study population (long-acting beta2-agonist (LABA) users vs. nonusers) 11](#_Toc193194924)

[**Supplementary Figure 1.** Flow chart of the study population selection**. 15**](#_Toc193194925)

[**Supplementary Figure 2.** Distributions of propensity scores between inhaled corticosteroid (ICS) users and nonusers: before and after propensity score matching 16](#_Toc193194926)

[**References** 17](#_Toc193194927)

**Supplementary Table 1. Definitions of acute asthma exacerbations**

| **Component** | **Definition** |
| --- | --- |
| Asthma-related ED visit | 1. Main diagnosis with asthma^1^ or any diagnosis with acute asthma exacerbation 2. Consider codes within ±1 day as 1 event 3. No hospital admission within ±1 day |
| Asthma-related hospitalization | Main diagnosis with asthma^2^ or any diagnosis with acute asthma exacerbation |
| Course of oral corticosteroids | 1. Prescription of prednisolone or methylprednisolone:    1. Outpatient database: ≥20 mg prednisolone equivalents per day for a duration of 3 to 27 days ^3^    2. Inpatient database: ≥60 mg prednisolone equivalents during each hospitalization 2. With asthma code within ±14 days of dispensing date |
| Abbreviations: ED, emergency department. | |

**Supplementary Table 2. Baseline characteristics of the study population (inhaled corticosteroid (ICS) users vs. nonusers)**

|  | **Before PSM (*N*=4,538)** | | | **After PSM (N=2,164)** | | |
| --- | --- | --- | --- | --- | --- | --- |
|  | **ICS users (*N*=1,713)** | **ICS nonusers (*N*=2,825)** | **aSMD** ^b^ | **ICS users (*N*=1,082)** | **ICS nonusers (N=1,082)** | **aSMD** ^b^ |
| **Sociodemographics** | | | | | | |
| **Age, N**^a^ **(%), years** | | | | | | |
| 18–19 | 29 (1.7) | 52 (1.8) | 0.09 | 18 (1.7) | 16 (1.5) | 0.08 |
| 20–34 | 1123 (65.6) | 1975 (69.9) |  | 735 (67.9) | 742 (68.6) |  |
| ≥35 | 561 (32.7) | 798 (28.2) |  | 329 (30.4) | 324 (29.9) |  |
| **Area of residence at delivery, N**^a^ **(%)** | | | | | | |
| Northern | 1,034 (60.4) | 1,720 (60.9) | 0.03 | 639 (59.1) | 637 (58.9) | 0.03 |
| Central | 324 (18.9) | 538 (19.0) |  | 210 (19.4) | 199 (18.4) |  |
| Southern | 305 (17.8) | 494 (17.5) |  | 193 (17.8) | 202 (18.7) |  |
| Eastern and offshore islands | 50 (2.9) | 73 (2.6) |  | 40 (3.7) | 44 (4.1) |  |
| **Average insurance premium, N**^a^ **(%), NT$ per month** | | | | | | |
| <20,000 | 488 (28.5) | 783 (27.7) | 0.05 | 296 (27.4) | 305 (28.2) | 0.03 |
| 20,000–29,999 | 610 (35.6) | 1,037 (36.7) |  | 385 (35.6) | 380 (35.1) |  |
| 30,000–39,999 | 244 (14.2) | 433 (15.3) |  | 155 (14.3) | 154 (14.2) |  |
| ≥40000 | 371 (21.7) | 572 (20.2) |  | 246 (22.7) | 243 (22.5) |  |
| **Neonate gender, N**^a^ **(%), male** | 870 (50.8) | 1,494 (52.9) | 0.04 | 548 (50.6) | 552 (51.0) | 0.01 |
| **Delivery year, N**^a^ **(%)** | | | | | | |
| 2009 | 213 (12.4) | 421 (14.9) | **0.19** | 146 (13.5) | 153 (14.1) | 0.07 |
| 2010 | 209 (12.2) | 222 (7.9) |  | 140 (12.9) | 145 (13.4) |  |
| 2011 | 193 (11.3) | 320 (11.3) |  | 120 (11.1) | 130 (12.0) |  |
| 2012 | 220 (12.8) | 340 (12.0) |  | 129 (11.9) | 124 (11.5) |  |
| 2013 | 186 (10.9) | 323 (11.4) |  | 116 (10.7) | 113 (10.4) |  |
| 2014 | 182 (10.6) | 275 (9.7) |  | 106 (9.8) | 99 (9.1) |  |
| 2015 | 176 (10.3) | 303 (10.7) |  | 106 (9.8) | 107 (9.9) |  |
| 2016 | 164 (9.6) | 355 (12.6) |  | 110 (10.2) | 110 (10.2) |  |
| 2017 | 170 (9.9) | 266 (9.4) |  | 109 (10.1) | 101 (9.3) |  |
| **Asthma-related variables** | | | | | | |
| **Asthma severity, N**^a,c^ **(%)** | | | | | | |
| Mild | 1,079 (63.0) | 2,788 (98.7) | **1.04** | 1,073 (99.2) | 1,076 (99.4) | 0.03 |
| Moderate | 386 (22.5) | 0 (0.0) |  | 0 (0.0) | 0 (0.0) |  |
| Severe | 248 (14.5) | 37 (1.3) |  | 9 (0.8) | 6 (0.6) |  |
| **Asthma exacerbations, N (%)** | | | | | | |
| Emergency visits | 42 (2.5) | 37 (1.3) | 0.08 | 12 (1.1) | 13 (1.2) | 0.01 |
| Hospitalizations | 5 (0.3) | 9 (0.3) | 0.01 | 1–3 (0.1–0.3) | 1-3 (0.1–0.3) | 0.03 |
| OCS use | 68 (4.0) | 129 (4.6) | 0.03 | 22 (2.0) | 22 (2.0) | 0.00 |
| **Comorbidities, N**^a^ **(%)** | | | | | | |
| Allergic rhinitis | 801 (46.8) | 806 (28.5) | **0.38** | 399 (36.9) | 390 (36.0) | 0.02 |
| Atopic dermatitis | 36 (2.1) | 54 (1.9) | 0.01 | 22 (2.0) | 18 (1.7) | 0.03 |
| Hypertension | 30 (1.8) | 50 (1.8) | 0.00 | 16 (1.5) | 13 (1.2) | 0.02 |
| Diabetes mellitus | 23 (1.3) | 50 (1.8) | 0.04 | 16 (1.5) | 12 (1.1) | 0.03 |
| Dyslipidemia | 21 (1.2) | 38 (1.3) | 0.01 | 13 (1.2) | 5 (0.5) | 0.08 |
| Cardiovascular disease | 11 (0.6) | 22 (0.8) | 0.02 | 6 (0.6) | 4 (0.4) | 0.03 |
| Depression | 48 (2.8) | 64 (2.3) | 0.03 | 27 (2.5) | 27 (2.5) | 0.00 |
| Anxiety | 79 (4.6) | 116 (4.1) | 0.02 | 51 (4.7) | 53 (4.9) | 0.01 |
| Bipolar disease | 8 (0.5) | 12 (0.4) | 0.01 | 5(0.5) | 2 (0.2) | 0.05 |
| Schizophrenia | 22 (1.3) | 24 (0.8) | 0.04 | 15(1.4) | 12 (1.1) | 0.03 |
| Epilepsy | 1-3 (0.1-0.2) | 4 (0.1) | 0.01 | 1-3 (0.1-0.3) | 1-3 (0.1-0.3) | 0.00 |
| Thyroid disorder | 60 (3.5) | 123 (4.4) | 0.04 | 36 (3.3) | 31 (2.9) | 0.03 |
| Renal disease | 1-3 (0.1-0.2) | 0 (0.0) | 0.03 | 0 (0.0) | 0 (0.0) | 0.00 |
| Anemia | 83 (4.8) | 108 (3.8) | 0.05 | 50 (4.6) | 50 (4.6) | 0.00 |
| Antiphospholipid syndrome | 1-3 (0.1-0.2) | 0 (0.0) | 0.03 | 0 (0.0) | 0 (0.0) | 0.00 |
| Obesity | 10 (0.6) | 14 (0.5) | 0.01 | 4 (0.4) | 1-3 (0.1-0.3) | 0.02 |
| Alcohol use | 10 (0.6) | 10 (0.4) | 0.03 | 7 (0.6) | 5 (0.5) | 0.02 |
| Drug dependence | 6 (0.4) | 5 (0.2) | 0.03 | 1–3 (0.1–0.3) | 1–3 (0.1–0.3) | 0.02 |
| **Medications** | | | | | | |
| **Anti-asthmatics, N**^a^ **(%)** | | | | | | |
| Relievers | 268 (15.6) | 237 (8.4) | **0.22** | 84 (7.8) | 90 (8.3) | 0.02 |
| ICSs | 630 (36.8) | 0 (0.0) | **1.08** | 0 (0.0) | 0 (0.0) | 0.00 |
| LABAs | 514 (30.0) | 0 (0.0) | **0.93** | 0 (0.0) | 0 (0.0) | 0.00 |
| LTRAs | 123 (7.2) | 95 (3.4) | **0.17** | 20 (1.8) | 15 (1.4) | 0.04 |
| Theophylline | 93 (5.4) | 44 (1.6) | **0.21** | 17 (1.6) | 14 (1.3) | 0.02 |
| OCSs | 83 (4.8) | 37 (1.3) | **0.21** | 9 (0.8) | 6 (0.6) | 0.03 |
| Omalizumab | 1–3 (0.1–0.2) | 0 (0.0) | 0.06 | 0 (0.0) | 0 (0.0) | 0.00 |
| Systemic corticosteroids ^d^ | 217 (12.7) | 376 (13.3) | 0.02 | 95 (8.8) | 92 (8.5) | 0.01 |
| Topical corticosteroids | 415 (24.2) | 526 (18.6) | **0.14** | 193 (17.8) | 180 (16.6) | 0.03 |
| NSAIDs | 571 (33.3) | 1,106 (39.2) | **0.12** | 358 (33.1) | 353 (32.6) | 0.01 |
| Antihistamines | 746 (43.5) | 1,245 (44.1) | 0.01 | 341 (31.5) | 344 (31.8) | 0.01 |
| Decongestants | 371 (21.7) | 631 (22.3) | 0.02 | 182 (16.8) | 173 (16.0) | 0.02 |
| Methylxanthines ^e^ | 271 (15.8) | 488 (17.3) | 0.04 | 125 (11.6) | 128 (11.8) | 0.01 |
| Antihypertensives | 35 (2.0) | 69 (2.4) | 0.03 | 23 (2.1) | 20 (1.8) | 0.02 |
| Antidiabetics | 16 (0.9) | 27 (1.0) | 0.00 | 9 (0.8) | 7 (0.6) | 0.02 |
| Antihyperlipidemics | 1–3 (0.1–0.2) | 11 (0.4) | 0.04 | 1–3 (0.1–0.3) | 1–3 (0.1–0.3) | 0.02 |
| Antithrombotics | 10 (0.6) | 22 (0.8) | 0.02 | 5 (0.5) | 7 (0.6) | 0.03 |
| Antidepressants | 57 (3.3) | 85 (3.0) | 0.02 | 32 (3.0) | 34 (3.1) | 0.01 |
| Benzodiazepines | 127 (7.4) | 262 (9.3) | 0.07 | 70 (6.5) | 72 (6.7) | 0.01 |
| Antipsychotics | 60 (3.5) | 85 (3.0) | 0.03 | 40 (3.7) | 37 (3.4) | 0.02 |
| Anticonvulsants | 24 (1.4) | 52 (1.8) | 0.04 | 16 (1.5) | 17 (1.6) | 0.01 |
| Antithyroid or thyroid preparations | 12 (0.7) | 40 (1.4) | 0.07 | 7 (0.6) | 7 (0.6) | 0.00 |
| Opioids | 23 (1.3) | 43 (1.5) | 0.02 | 15 (1.4) | 10 (0.9) | 0.04 |
| Codeine | 6 (0.4) | 11 (0.4) | 0.01 | 1-3 (0.1-0.3) | 4 (0.4) | 0.04 |
| Antibiotics | 551 (32.2) | 1,041 (36.8) | 0.10 | 318 (29.4) | 325 (30.0) | 0.01 |
| Folic acid | 4 (0.2) | 7 (0.2) | 0.00 | 1-3 (0.1-0.3) | 1-3 (0.1-0.3) | 0.02 |
| Abbreviations: aSMD, absolute standardized mean difference; LABAs, long-acting beta2 agonists; LTRAs, leukotriene receptor antagonists; NSAIDs, non-steroidal anti-inflammatory drugs; OCSs, oral corticosteroids; PSM, propensity score matching.  ^a^ 1≤*n≤*3 was displayed as the range due to personal privacy concerns.  ^b^ aSMD >0.1 is shown in bold format.  ^c^ Asthma severity categorized by stepwise treatment: mild (steps 0–2), moderate (step 3), severe (steps 4–5).  ^d^ OCSs used for asthma exacerbations or acute exacerbations were not included.  ^e^ Theophylline used for asthma exacerbations was not included. | | | | | | |

**Supplementary Table 3. Baseline characteristics of the study population (high-dose vs. low-to-moderate-dose inhaled corticosteroids (ICSs))**

|  | **Before IPTW (*N*=800)** | | | **After IPTW (*N*=789.8)** | | |
| --- | --- | --- | --- | --- | --- | --- |
|  | **High-dose ICS (*N*=37)** | **Low-to-moderate-dose ICS**  **(*N*=763)** | **aSMD**^b^ | **High-dose ICS (*N*=22.4)** | **Low-to-moderate-dose ICS**  **(*N*=767.3)** | **aSMD**^b^ |
| **Sociodemographics** | | | | | | |
| **Age, N**^a^ **(%), years** | | | | | | |
| 18–19 | 0 (0.0) | 6 (0.8) | **0.31** | 0.0 (0.0) | 5.7 (0.7) | **0.17** |
| 20–34 | 18 (48.6) | 472 (61.9) |  | 14.7 (65.6) | 465.3 (60.6) |  |
| ≥35 | 19 (51.4) | 285 (37.4) |  | 7.7 (34.4) | 296.3 (38.6) |  |
| **Area of residence at delivery, N**^a^ **(%)** | | | | | | |
| Northern | 24 (64.9) | 465 (60.9) | **0.43** | 13.9 (61.7) | 465.1 (60.6) | **0.48** |
| Central | 8 (21.6) | 138 (18.1) |  | 3.0 (13.5) | 143.0 (18.6) |  |
| Southern | 5 (13.5) | 141 (18.5) |  | 5.6 (24.7) | 141.1 (18.4) |  |
| Eastern and offshore islands | 0 (0.0) | 19 (2.5) |  | 0.0 (0.0) | 18.1 (2.4) |  |
| **Average insurance premium, N**^a^ **(%), NT$ per month** | | | | | | |
| <20,000 | 12 (32.4) | 213 (27.9) | **0.12** | 7.3 (32.7) | 218.2 (28.4) | **0.49** |
| 20,000–29,999 | 12 (32.4) | 277 (36.3) |  | 4.2 (18.8) | 277.3 (36.1) |  |
| 30,000–39,999 | 6 (16.2) | 109 (14.3) |  | 2.0 (9.1) | 108.3 (14.1) |  |
| ≥40,000 | 7 (18.9) | 164 (21.5) |  | 8.8 (39.4) | 163.5 (21.3) |  |
| **Neonate gender, N**^a^ **(%), male** | 22 (59.5) | 402 (52.7) | **0.14** | 17.1 (76.0) | 403.2 (52.5) | **0.51** |
| **Delivery year, N**^a^ **(%)** | | | | | | |
| 2009 | 1–3  (2.7–8.1) | 82 (10.7) | **0.48** | 3.2 (14.3) | 81.8 (10.7) | **0.88** |
| 2010 | 4 (10.8) | 92 (12.1) |  | 5.0 (22.3) | 92.0 (12.0) |  |
| 2011 | 7 (18.9) | 70 (9.2) |  | 0.8 (3.4) | 72.1 (9.4) |  |
| 2012 | 6 (16.2) | 97 (12.7) |  | 1.4 (6.4) | 97.2 (12.7) |  |
| 2013 | 4 (10.8) | 89 (11.7) |  | 2.4 (10.9) | 88.1 (11.5) |  |
| 2014 | 1–3  (2.7–8.1) | 84 (11.0) |  | 1.3 (6.0) | 88.5 (11.5) |  |
| 2015 | 1–3  (2.7–8.1) | 92 (12.1) |  | 0.3 (1.5) | 88.6 (11.6) |  |
| 2016 | 5 (13.5) | 80 (10.5) |  | 1.6 (6.9) | 81.2 (10.6) |  |
| 2017 | 4 (10.8) | 77 (10.1) |  | 6.4 (28.4) | 77.8 (10.1) |  |
| **Asthma-related variables** | | | | | | |
| **Asthma severity, N**^a,c^ **(%)** | | | | | | |
| Mild | 6 (16.2) | 147 (19.3) | **0.98** | 13.2 (58.6) | 388.2 (50.6) | **0.20** |
| Moderate | 10 (27.0) | 215 (28.2) |  | 4.4 (19.6) | 215.1 (28.0) |  |
| Severe | 21 (56.8) | 401 (52.6) |  | 4.9 (21.8) | 164.1 (21.4) |  |
| **Asthma exacerbations, N**^a^ **(%)** | | | | | | |
| Emergency visits | 1–3  (2.7–8.1) | 19 (2.5) | **0.25** | 0.3 (1.2) | 25.1 (3.3) | **0.14** |
| Hospitalization | 1–3  (2.7–8.1) | 1-3  (0.1-0.4) | **0.33** | 0.2 (0.9) | 7.4 (1.0) | 0.01 |
| OCS use | 4 (10.8) | 32 (4.2) | **0.25** | 0.4 (2.0) | 38.3 (5.0) | **0.17** |
| **Comorbidities, N**^a^ **(%)** | | | | | | |
| Allergic rhinitis | 23 (62.2) | 400 (52.4) | **0.20** | 9.3 (41.2) | 404.2 (52.7) | **0.23** |
| Atopic dermatitis | 0 (0.0) | 15 (2.0) | **0.20** | 0.0 (0.0) | 14.3 (1.9) | **0.20** |
| Hypertension | 1–3  (2.7–8.1) | 17 (2.2) | 0.03 | 0.1 (0.3) | 16.4 (2.1) | **0.17** |
| Diabetes mellitus | 1–3  (2.7–8.1) | 11 (1.4) | 0.09 | 0.1 (0.3) | 10.7 (1.4) | **0.13** |
| Dyslipidemia | 0 (0.0) | 10 (1.3) | **0.16** | 0.0 (0.0) | 9.5 (1.2) | **0.16** |
| Cardiovascular disease | 1–3  (2.7–8.1) | 4 (0.5) | **0.17** | 0.1 (0.3) | 4.0 (0.5) | 0.04 |
| Depression | 1–3  (2.7–8.1) | 20 (2.6) | **0.14** | 0.3 (1.3) | 22.0 (2.9) | **0.11** |
| Anxiety | 1–3  (2.7–8.1) | 37 (4.8) | **0.13** | 1.5 (6.9) | 40.0 (5.2) | 0.07 |
| Bipolar disease | 0 (0.0) | 1-3  (0.1-0.4) | 0.09 | 0.0 (0.0) | 2.9 (0.4) | 0.09 |
| Schizophrenia | 0 (0.0) | 9 (1.2) | **0.16** | 0.0 (0.0) | 8.6 (1.1) | **0.15** |
| Thyroid disorder | 0 (0.0) | 28 (3.7) | **0.28** | 0.0 (0.0) | 26.7 (3.5) | **0.27** |
| Anemia | 1–3  (2.7–8.1) | 42 (5.5) | **0.10** | 0.8 (3.4) | 42.3 (5.5) | **0.11** |
| Antiphospholipid syndrome | 0 (0.0) | 1-3 (0.1-0.4) | 0.05 | 0.0 (0.0) | 1.0 (0.1) | 0.05 |
| Obesity | 0 (0.0) | 7 (0.9) | **0.14** | 0.0 (0.0) | 6.7 (0.9) | **0.13** |
| Alcohol use | 0 (0.0) | 5 (0.7) | **0.12** | 0.0 (0.0) | 4.8 (0.6) | **0.11** |
| Drug dependence | 0 (0.0) | 1-3 (0.1-0.4) | 0.05 | 0.0 (0.0) | 1.0 (0.1) | 0.05 |
| **Medications, N**^a^ **(%)** | | | | | | |
| **Antiasthmatics** | | | | | | |
| Relievers | 9 (24.3) | 136 (17.8) | **0.16** | 3.0 (13.5) | 142.9 (18.6) | **0.14** |
| Mean ICS dose | | | | | | |
| 0 | 6 (16.2) | 405 (53.1) | **1.18** | 13.2 (58.6) | 389.1 (50.7) | **0.42** |
| Less than low | 0 (0.0) |  |  | 0.0 (0.0) | 2.9 (0.4) |  |
| Low | 4 (10.8) | 158 (20.7) |  | 1.8 (8.1) | 153.5 (20.0) |  |
| Medium | 13 (35.1) | 160 (21.0) |  | 4.2 (18.9) | 169.0 (22.0) |  |
| High | 14 (37.8) | 40 (5.2) |  | 3.2 (14.4) | 52.9 (6.9) |  |
| LABAs | 26 (70.3) | 303 (39.7) | **0.65** | 7.8 (34.7) | 318.5 (41.5) | **0.14** |
| LTRAs | 10 (27.0) | 69 (9.0) | **0.48** | 2.5 (11.1) | 76.2 (9.9) | 0.04 |
| Theophylline | 1-3 (2.7-8.1) | 49 (6.4) | 0.04 | 1.2 (5.5) | 48.3 (6.3) | 0.04 |
| OCSs | 1-3 (2.7-8.1) | 38 (5.0) | 0.02 | 0.8 (3.6) | 38.3 (5.0) | 0.07 |
| Omalizumab | 1-3 (2.7-8.1) | 1-3 (0.1-0.4) | **0.20** | 0.1 (0.3) | 2.2 (0.3) | 0.00 |
| Systemic corticosteroids ^d^ | 6 (16.2) | 99 (13.0) | 0.09 | 1.2 (5.4) | 104.1 (13.6) | **0.28** |
| Topical corticosteroids | 12 (32.4) | 209 (27.4) | **0.11** | 3.9 (17.5) | 210.0 (27.4) | **0.24** |
| NSAIDs | 12 (32.4) | 254 (33.3) | 0.02 | 5.6 (25.1) | 254.0 (33.1) | **0.18** |
| Antihistamines | 16 (43.2) | 355 (46.5) | 0.07 | 4.6 (20.6) | 353.6 (46.1) | **0.56** |
| Decongestants | 9 (24.3) | 171 (22.4) | 0.05 | 1.8 (7.9) | 170.6 (22.2) | **0.41** |
| Methylxanthines ^e^ | 7 (18.9) | 119 (15.6) | 0.09 | 0.9 (4.0) | 123.5 (16.1) | **0.41** |
| Antihypertensives | 1–3 (2.7–8.1) | 12 (1.6) | 0.08 | 0.1 (0.3) | 11.7 (1.5) | **0.14** |
| Antidiabetics | 0 (0.0) | 8 (1.0) | **0.15** | 0.0 (0.0) | 7.6 (1.0) | **0.14** |
| Antihyperlipidemics | 0 (0.0) | 1–3 (0.1–0.4) | 0.05 | 0.0 (0.0) | 1.0 (0.1) | 0.05 |
| Antithrombotics | 0 (0.0) | 8 (1.0) | **0.15** | 0.0 (0.0) | 7.6 (1.0) | **0.14** |
| Antidepressants | 1–3 (2.7–8.1) | 27 (3.5) | 0.09 | 0.3 (1.3) | 28.7 (3.7) | **0.16** |
| Benzodiazepines | 4 (10.8) | 59 (7.7) | **0.11** | 0.7 (3.2) | 65.1 (8.5) | **0.23** |
| Antipsychotics | 1–3 (2.7–8.1) | 26 (3.4) | 0.04 | 0.1 (0.6) | 25.5 (3.3) | **0.20** |
| Anticonvulsants | 0 (0.0) | 11 (1.4) | **0.17** | 0.0 (0.0) | 10.5 (1.4) | **0.17** |
| Antithyroid or thyroid preparations | 0 (0.0) | 4 (0.5) | **0.10** | 0.0(0.0) | 3.8 (0.5) | **0.10** |
| Opioids | 0 (0.0) | 12 (1.6) | **0.18** | 0.0 (0.0) | 11.4 (1.5) | **0.17** |
| Codeine | 0 (0.0) | 4 (0.5) | **0.10** | 0.0 (0.0) | 3.8 (0.5) | **0.10** |
| Antibiotics | 11 (29.7) | 252 (33.0) | 0.07 | 3.2 (14.1) | 249.5 (32.5) | **0.45** |
| Folic acid | 0 (0.0) | 1-3 (0.1-0.4) | 0.07 | 0.0 (0.0) | 1.9 (0.2) | 0.07 |
| Abbreviations: aSMD, absolute standardized mean difference; IPTW, inverse probability of treatment weighting; LABAs, long-acting beta2 agonists; LTRAs, leukotrienes receptor antagonists; NSAIDs, non-steroidal anti-inflammatory drugs; OCSs oral corticosteroids.  ^a^ 1≤*n≤*3 was displayed in the range or merged cells due to personal privacy concerns.  ^b^ aSMD >0.1 is shown in bold format.  ^c^ Asthma severity categorized by stepwise treatment: mild (steps 0–2), moderate (step 3), severe (steps 4–5).  ^d^ OCSs used for asthma exacerbations or acute exacerbations were not included.  ^e^ Theophylline used for asthma exacerbations was not included. | | | | | | |

**Supplementary Table 4. Baseline characteristics of the study population (long-acting beta2-agonist (LABA) users vs. nonusers)**

|  | **Before PSM (*N*=1,686)** | | | **After PSM (*N*=914)** | | |
| --- | --- | --- | --- | --- | --- | --- |
|  | **LABA users (*N*=1,207)** | **LABA nonusers (*N*=479)** | **aSMD**^b^ | **LABA users (*N*=457)** | **LABA nonusers (*N*=457)** | **aSMD**^b^ |
| **Sociodemographics** | | | | | | |
| **Age, N**^a^ **(%), years** | | | | | | |
| 18–19 | 22 (1.8) | 7 (1.5) | 0.09 | 14 (3.1) | 7 (1.5) | **0.10** |
| 20–34 | 795 (65.9) | 309 (64.5) |  | 313 (68.5) | 295 (64.6) |  |
| ≥35 | 390 (32.3) | 163 (34.0) |  | 130 (28.4) | 155 (33.9) |  |
| **Area of residence at delivery, N**^a^ **(%**)**)** | | | | | | |
| Northern | 693 (57.4) | 319 (66.6) | **0.24** | 182 (39.8) | 301 (65.9) | **0.60** |
| Central | 244 (20.2) | 78 (16.3) |  | 125 (27.4) | 77 (16.8) |  |
| Southern | 240 (19.9) | 62 (12.9) |  | 140 (30. 6) | 59 (12.9) |  |
| Eastern and offshore islands | 30 (2.5) | 20 (4.2) |  | 10 (2.2) | 20 (4.4) |  |
| **Average insurance premium, N**^a^ **(%), NT% per month** | | | | | | |
| <20,000 | 352 (29.2) | 128 (26.7) | 0.07 | 149 (32.6) | 119 (26.0) | **0.17** |
| 20,000–29,999 | 435 (36.0) | 166 (34.7) |  | 161 (35.2) | 163 (35.7) |  |
| 30,000–39,999 | 166 (13.8) | 72 (15.0) |  | 59 (12.9) | 68 (14.9) |  |
| ≥40,000 | 254 (21.0) | 113 (23.6) |  | 88 (19.3) | 107 (23.4) |  |
| **Neonate gender, N**^a^ **(%), male** | 606 (50.2) | 252 (52.6) | 0.05 | 233 (51.0) | 238 (52.1) | 0.02 |
| **Delivery year, N**^a^ **(%)** | | | | | | |
| 2009 | 137 (11.4) | 73 (15.2) | **0.22** | 43 (9.4) | 69 (15.1) | **0.42** |
| 2010 | 152 (12.6) | 53 (11.1) |  | 82 (17.9) | 51 (11.2) |  |
| 2011 | 128 (10.6) | 60 (12.5) |  | 30 (6.6) | 57 (12.5) |  |
| 2012 | 163 (13.5) | 51 (10.6) |  | 74 (16.2) | 49 (10.7) |  |
| 2013 | 122 (10.1) | 61 (12.7) |  | 35 (7.7) | 56 (12.3) |  |
| 2014 | 132 (10.9) | 48 (10.0) |  | 54 (11.8) | 45 (9.8) |  |
| 2015 | 124 (10.3) | 51 (10.6) |  | 32 (7.0) | 48 (10.5) |  |
| 2016 | 119 (9.9) | 44 (9.2) |  | 45 (9.8) | 44 (9.6) |  |
| 2017 | 130 (10.8) | 38 (7.9) |  | 62 (13.6) | 38 (8.3) |  |
| **Asthma-related variables** | | | | | | |
| **Asthma severity, N**^a,c^ **(%)** | | | | | | |
| Mild | 699 (57.9) | 380 (79.3) | **0.49** | 443 (96.9) | 378 (82.7) | **0.48** |
| Moderate | 290 (24.0) | 74 (15.4) |  | 14 (3.1) | 64 (14.0) |  |
| Severe | 218 (18.1) | 25 (5.2) |  |  | 15 (3.3) |  |
| **Asthma exacerbations, N**^a^ **(%)** | | | | | | |
| Emergency visits | 33 (2.7) | 8 (1.7) | 0.07 | 4 (0.9) | 6 (1.3) | 0.04 |
| Hospitalization | 5 (0.4) | 0 (0.0) | 0.09 | 0 (0.0) | 0 (0.0) | 0.00 |
| OCS use | 44 (3.6) | 22 (4.6) | 0.05 | 6 (1.3) | 17 (3.7) | **0.15** |
| **Comorbidities, N**^a^ **(%)** | | | | | | |
| Allergic rhinitis | 594 (49.2) | 188 (39.2) | **0.20** | 203 (44.4) | 176 (38.5) | **0.12** |
| Atopic dermatitis | 25 (2.1) | 10 (2.1) | 0.00 | 15 (3.3) | 9 (2.0) | 0.08 |
| Hypertension | 23 (1.9) | 6 (1.3) | 0.05 | 9 (2.0) | 5 (1.1) | 0.07 |
| Diabetes mellitus | 14 (1.2) | 8 (1.7) | 0.04 | 5 (1.1) | 8 (1.8) | 0.06 |
| Dyslipidemia | 14 (1.2) | 6 (1.3) | 0.01 | 6 (1.3) | 6 (1.3) | 0.00 |
| Cardiovascular disease | 9 (0.7) | 1–3  (0.2–0.6) | 0.04 | 1–3  (0.2–0.7) | 1–3  (0.2–0.7) | 0.00 |
| Depression | 36 (3.0) | 12 (2.5) | 0.03 | 15 (3.3) | 12 (2.6) | 0.04 |
| Anxiety | 63 (5.2) | 16 (3.3) | 0.09 | 38 (8.3) | 15 (3.3) | **0.22** |
| Bipolar disease | 7 (0.6) | 1–3  (0.2–0.6) | 0.06 | 1–3  (0.2–0.7) | 1–3  (0.2–0.7) | 0.07 |
| Schizophrenia | 14 (1.2) | 8 (1.7) | 0.04 | 8 (1.8) | 8 (1.8) | 0.00 |
| Epilepsy | 1–3 (0.1–0.2) | 0 (0.0) | 0.06 | 0 (0.0) | 0 (0.0) | 0.00 |
| Thyroid disorder | 40 (3.3) | 19 (4.0) | 0.04 | 20 (4.4) | 18 (3.9) | 0.02 |
| Renal disease | 1–3 (0.1–0.2) | 0 (0.0) | 0.04 | 0 (0.0) | 0 (0.0) | 0.00 |
| Anemia | 59 (4.9) | 22 (4.6) | 0.01 | 31 (6.8) | 20 (4.4) | **0.11** |
| Antiphospholipid syndrome | 1–3 (0.1–0.2) | 0 (0.0) | 0.04 | 0 (0.0) | 0 (0.0) | 0.00 |
| Obesity | 9 (0.7) | 1–3  (0.2–0.6) | 0.08 | 1–3  (0.2–0.7) | 1–3  (0.2–0.7) | 0.07 |
| Alcohol use | 9 (0.7) | 1–3  (0.2–0.6) | 0.08 | 5 (1.1) | 1–3  (0.2–0.7) | **0.11** |
| Drug dependence | 5 (0.4) | 1–3  (0.2–0.6) | 0.04 | 1–3  (0.2–0.7) | 1–3  (0.2–0.7) | 0.04 |
| **Medications, N**^a^ **(%)** | | | | | | |
| **Anti-asthmatics** | | | | | | |
| Relievers | 190 (15.7) | 71 (14.8) | 0.03 | 35 (7.7) | 62 (13.6) | **0.19** |
| Mean ICS dose | | | | | | |
| 0 | 704 (58.3) | 382 (79.7) | **0.47** | 443 (96.9) | 379 (82.9) | **0.49** |
| Less than low | 9 (0.7) |  |  | 0 (0.0) | 0 (0.0) |  |
| Low | 246 (20.4) | 43 (9.0) |  | 7 (1.5) | 39 (8.5) |  |
| Medium | 199 (16.5) | 41 (8.6) |  | 7 (1.5) | 28 (6.1) |  |
| High | 49 (4.1) | 13 (2.7) |  |  | 11 (2.4) |  |
| LABAs | 487 (40.3) | 0 (0.0) | **1.16** | 0 (0.0) | 0 (0.0) | 0.00 |
| LTRAs | 103 (8.5) | 14 (2.9) | **0.24** | 7 (1.5) | 11 (2.4) | 0.06 |
| Theophylline | 69 (5.7) | 21 (4.4) | 0.06 | 5 (1.1) | 15 (3.3) | **0.15** |
| OCSs | 62 (5.1) | 19 (4.0) | 0.06 | 3 (0.7) | 15 (3.3) | **0.19** |
| Omalizumab | 1-3 (0.1-0.2) | 0 (0.0) | 0.07 | 0 (0.0) | 0 (0.0) | 0.00 |
| Systemic corticosteroids ^d^ | 171 (14.2) | 42 (8.8) | **0.17** | 50 (10.9) | 39 (8.5) | 0.08 |
| Topical corticosteroids | 308 (25.5) | 98 (20.5) | **0.12** | 87 (19.0) | 93 (20.4) | 0.03 |
| NSAIDs | 391 (32.4) | 169 (35.3) | 0.06 | 156 (34.1) | 160 (35.0) | 0.02 |
| Antihistamines | 553 (45.8) | 180 (37.6) | **0.17** | 162 (35.4) | 165 (36.1) | 0.01 |
| Decongestants | 268 (22.2) | 95 (19.8) | 0.06 | 74 (16.2) | 83 (18.2) | 0.05 |
| Methylxanthines ^e^ | 195 (16.2) | 71 (14.8) | 0.04 | 55 (12.0) | 64 (14.0) | 0.06 |
| Antihypertensives | 21 (1.7) | 12 (2.5) | 0.05 | 5 (1.1) | 12 (2.6) | **0.11** |
| Antidiabetics | 10 (0.8) | 4 (0.8) | 0.00 | 1–3  (0.2–0.7) | 4 (0.9) | 0.09 |
| Antihyperlipidemics | 1–3  (0.1–0.2) | 0 (0.0) | 0.06 | 0 (0.0) | 0 (0.0) | 0.00 |
| Antithrombotics | 6 (0.5) | 4 (0.8) | 0.04 | 1–3  (0.2–0.7) | 1–3  (0.2–0.7) | 0.03 |
| Antidepressants | 46 (3.8) | 11 (2.3) | 0.09 | 21 (4.6) | 11 (2.4) | **0.12** |
| Benzodiazepines | 96 (8.0) | 31 (6.5) | 0.06 | 34 (7.4) | 29 (6.3) | 0.04 |
| Antipsychotics | 42 (3.5) | 18 (3.8) | 0.02 | 13 (2.8) | 17 (3.7) | 0.05 |
| Anticonvulsants | 16 (1.3) | 8 (1.7) | 0.03 | 6 (1.3) | 7 (1.5) | 0.02 |
| Antithyroid or thyroid preparations | 6 (0.5) | 6 (1.3) | 0.08 | 1–3  (0.2–0.7) | 5 (1.1) | 0.08 |
| Opioids | 17 (1.4) | 6 (1.3) | 0.01 | 6 (1.3) | 6 (1.3) | 0.00 |
| Codeine | 6 (0.5) | 0 (0.0) | **0.10** | 0 (0.0) | 0 (0.0) | 0.00 |
| Antibiotics | 386 (32.0) | 156 (32.6) | 0.01 | 132 (28.9) | 147 (32.2) | 0.07 |
| Folic acid | 4 (0.3) | 0 (0.0 | 0.08 | 0 (0.0) | 0 (0.0) | 0.00 |
| Abbreviations: aSMD, absolute standardized mean difference; ICSs, inhaled corticosteroids; LTRA, leukotriene receptor antagonists; NSAIDs, non-steroidal anti-inflammatory drugs; OCSs, oral corticosteroids; PSM, propensity score matching.  ^a^ 1≤*n*≤3 was displayed in range or merged cells due to personal privacy concerns.  ^b^ aSMD >0.1 is shown in bold format.  ^c^ Asthma severity categorized by stepwise treatment: mild (steps 0–2), moderate (step 3), severe (steps 4–5).  ^d^ OCSs used for asthma exacerbations or acute exacerbations were not included.  ^e^ Theophylline used for asthma exacerbations was not included. | | | | | | |

**Supplementary Figure 1. Flow chart of the study population selection.**

Supplementary Figure 1 illustrates the selection of the study population. Women exposed to ICS were further stratified based on their ICS dose and LABA exposure.


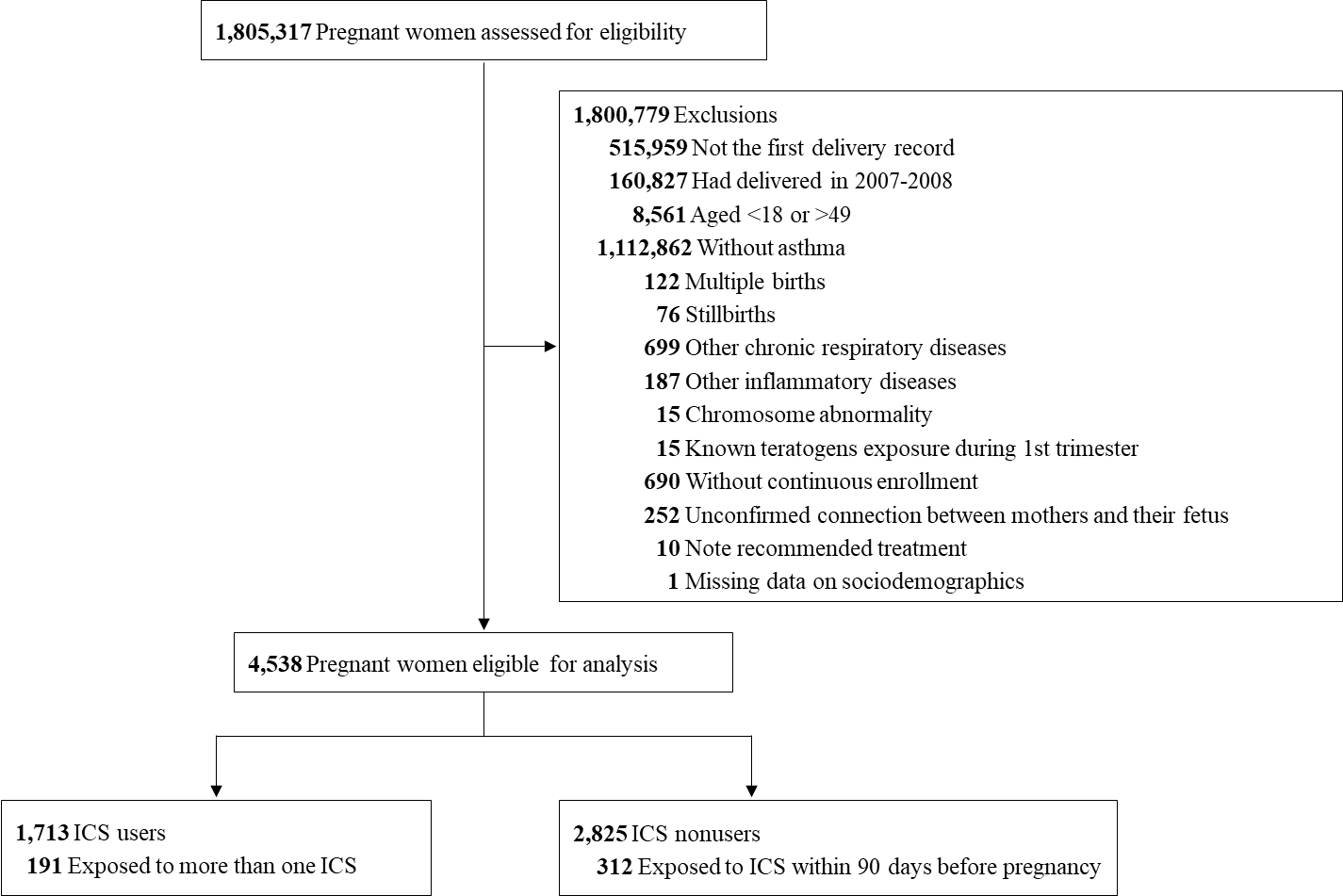


**Supplementary Figure 2.** **Distributions of propensity scores between inhaled corticosteroid (ICS) users and nonusers: before and after propensity score matching**

Supplementary Figure 2 presents the distributions of propensity scores of ICS users (dashed line) and ICS nonusers (solid line). The distributions of propensity scores between the two groups highly overlapped after propensity score matching.


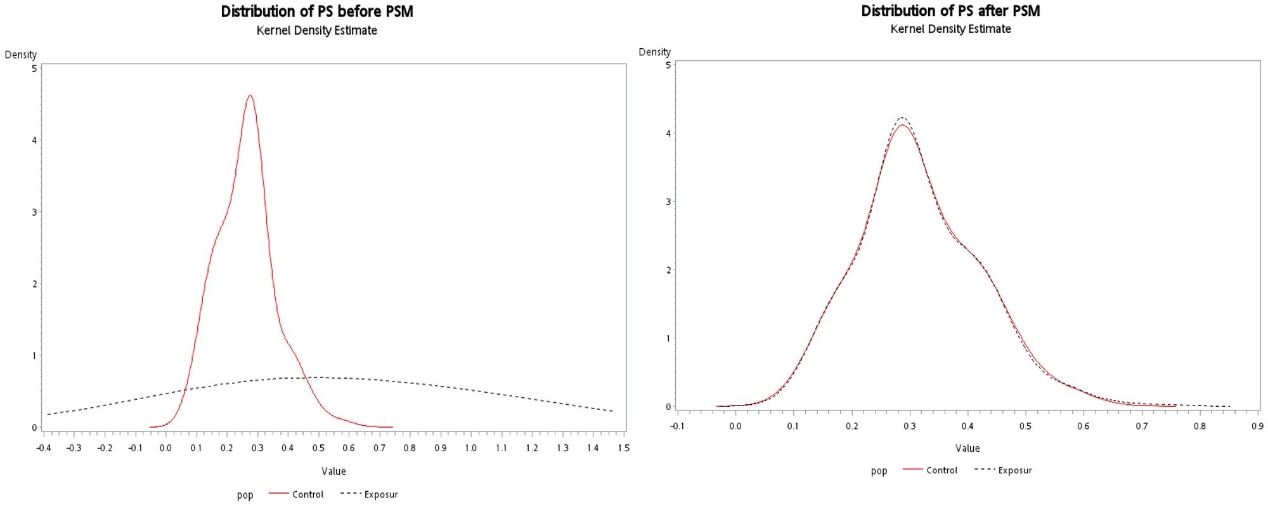


**References**

1. Kao YH, Wu SC. Effect of Continuity of Care on Emergency Department Visits in Elderly Patients with Asthma in Taiwan. *J Am Board Fam Med*. May-Jun 2017;30(3):384-395. doi:10.3122/jabfm.2017.03.160285

2. Yeh KW, Fang W, Huang JL. Increasing the hospitalization of asthma in children not in adults - from a national survey in Taiwan 1996-2002. *Pediatr Allergy Immunol*. Feb 2008;19(1):13-9. doi:10.1111/j.1399-3038.2007.00598.x

3. Global Initiative for Asthma. Global Strategy for Asthma Management and Prevention, 2022. [www.ginasthma.org](file:///D:\School\P3D\TMU\Projects\Student%20projects\Alice\新生兒氣喘\Submission\Respirology\Supplmentary\From%20Alice\www.ginasthma.org)
